# Effects of *AGXT2* variants on blood pressure and blood sugar among 750 older Japanese subjects recruited by the complete enumeration survey method

**DOI:** 10.1186/s12864-021-07612-3

**Published:** 2021-04-20

**Authors:** Yuta Yoshino, Hiroshi Kumon, Takaaki Mori, Taku Yoshida, Ayumi Tachibana, Hideaki Shimizu, Jun-ichi Iga, Shu-ichi Ueno

**Affiliations:** 1 Department of Neuropsychiatry, Molecules and Function, Ehime University Graduate School of Medicine, Shitsukawa, Toon, Ehime 791-0295 Japan; 2 Department of Neuropsychiatry, Zaidan Niihama Hospital, 13-47 Matsubara, Niihama, Ehime 792-0828 Japan

**Keywords:** AGXT2, ADMA, Blood pressure, Casual blood sugar

## Abstract

**Background:**

Alanine:glyoxylate aminotransferase 2 (AGXT2; EC 2.6.1.44) is the only enzyme that degrades the R-form of 3-aminoisobutyrate, an intermediate metabolite of thymine. AGXT2, as well as diaminoarginine dimethylaminohydrolase 1 (DDAH1; EC 3.5.3.18), works as an enzyme that degrades asymmetric dimethylarginine (ADMA), which competitively inhibits the nitric oxide synthase family. Thus, these two enzyme activities may change vascular vulnerability for a lifetime via the nitric oxide (NO) system. We investigated the association between vascular conditions and diseases such as hypertension and diabetes mellitus and polymorphisms of these two genes in 750 older Japanese subjects (mean age ± standard deviation, 77.0 ± 7.6 years) recruited using the complete enumeration survey method in the Nakayama study. Demographic and biochemical data, such as blood pressure (BP) and casual blood sugar (CBS), were obtained. Four functional single nucleotide polymorphisms (SNPs; rs37370, rs37369, rs180749, and rs16899974) of *AGXT2* and one functional insertion/deletion polymorphism in the promotor region with four SNPs (rs307894, rs669173, rs997251, and rs13373844) of *DDAH1* were investigated. Plasma ADMA was also analyzed in 163 subjects.

**Results:**

The results of multiple regression analysis showed that a loss of the functional haplotype of *AGXT2*, CAAA, was significantly positively correlated with BP (systolic BP, *p* = 0.034; diastolic BP, *p* = 0.025) and CBS (*p* = 0.021). No correlation was observed between *DDAH1* and either BP or CBS. ADMA concentrations were significantly elevated in subjects with two CAAA haplotypes compared with subjects without the CAAA haplotype (*p* = 0.033).

**Conclusions:**

Missense variants of *AGXT2*, but not *DDAH1*, may be related to vulnerability to vascular diseases such as hypertension and DM via the NO system.

**Supplementary Information:**

The online version contains supplementary material available at 10.1186/s12864-021-07612-3.

## Background

Cardiovascular disease (CVD) is one of the reasons for premature death, which is estimated from 4% in high-income countries to 42% in low-income countries [1]. The risk factor for developing CVD has been investigated but still unclear. Alanine:glyoxylate aminotransferase 2 (AGXT2; EC 2.6.1.44) is the only enzyme that has the ability to metabolize the R-form of 3-aminoisobutyrate (R-3-AIB) with pyruvate to 2-methyl-3-oxopropanoate and L-alanine [2]. Interestingly, AGXT2 activity depends on the individual’s genetic background; 30–40% of Japanese people show a lack of AGXT activity, compared with only 10% of individuals with European ancestry [2]. It has been shown that AGXT2 activity is decided by four functional single nucleotide polymorphisms (SNPs): rs37370 [3], rs37369 [4], rs180749 [5], and rs16899974 [6]. Furthermore, we previously found that the CAAA haplotype predicted by these four functional SNPs is strongly associated with loss of function in AGXT2 activity [7].

AGXT2 is also capable of metabolizing asymmetric dimethylarginine (ADMA), a unique methyl amino acid that competitively inhibits the nitric oxide synthase (NOS) family. ADMA is also metabolized by diaminoarginine dimethylaminohydrolase 1 (DDAH1; EC 3.5.3.18). Numerous studies have shown the association between serum/plasma ADMA levels and several diseases, including hypertension [8], congestive heart failure [9], chronic kidney disease [10], atherosclerosis [11], type 2 diabetes mellitus (DM) [12], as well as the human metabolome [3]. In addition, the variants of two enzymes have been shown to be associated with several vascular conditions and diseases (*AGXT2*: carotid atherosclerosis [7], atrial fibrillation and ischemic stroke [13], and coronary heart disease [14]; *DDAH1*: hypertension [15] and T2DM [16]).

Considering that *Agxt2* knockout (KO) mice show elevated levels of ADMA in circulation with reduced NO concentration and hypertension [17], it is possible that AGXT2 is a regulator of blood pressure (BP) even in humans. *AGXT2* is known to be expressed abundantly in the liver and kidney (RNA-seq data in GeneCards), and four functional SNPs of *AGXT2* may affect the liver and kidney functions of AGXT2 enzyme activity. To elucidate these points, we conducted the present study as follows. First, we determined the genotypes of four functional SNPs of *AGXT2*, as well as one functional polymorphism with four SNPs of *DDAH1*, in 750 subjects (aged > 65 years) recruited using the complete enumeration survey (census) method in the Nakayama study. Second, we tested whether plasma ADMA concentrations were regulated by *AGXT2* and/or *DDAH1*. Lastly, we examined whether the *AGXT2* and *DDAH1* genotypes were associated with clinical demographical and biological data such as BP and blood sugar using multiple regression analysis.

## Methods

### Subjects recruited from the Nakayama study

Nakayama is a rural community in Iyo city, Ehime Prefecture, Japan (2655 residents). This study was conducted with all older people aged > 65 years and living at home in Nakayama town. The subjects (927/1512, 61.3%) were recruited between January 2017 and April 2018. After excluding patients with dementia, 750 subjects were included in this study. Systolic blood pressure (SBP) and diastolic blood pressure (DBP) were measured three times, and the average values were used. All subjects were of unrelated Japanese origin and signed written informed consent forms approved by the institutional ethics committees of Ehime University Graduate School of Medicine (No. 1901009).

### Subjects in the urinary excretion of R-3-AIB and plasma ADMA concentration studies

Eighty-five unrelated Japanese subjects (41 males, 44 females; mean age = 48.3 ± 21.0 years) were recruited from Ehime Prefecture, Japan, to analyze the association between urinary excretion of R-3-AIB and the CAAC *AGXT2* haplotype [5, 7]. The cohort consisted of volunteer hospital workers, students, and citizens living in the same prefecture who did not have any urinary disorders as judged by blood examination. In addition to these 85 participants, 78 subjects were randomly selected from the Nakayama study to investigate whether ADMA concentrations were associated with four functional SNPs and the CAAA haplotype of *AGXT2*.

### Blood sampling and DNA isolation

Blood was collected into an EDTA tube. Subsequently, the plasma was collected as supernatant after centrifuging at 2000 rpm for 10 min and submitted to LSI Medience Corporation (Tokyo, Japan) to examine the biochemical data. Casual blood sugar (CBS) was judged based on the interview of eating habits and measured by the hexokinase/G6PD method. In addition, a blood cell component (remaining precipitation) was used for DNA isolation from whole blood leukocytes. Briefly, DNA was isolated from frozen white blood cells using a blood mini-kit with QIAcube (Qiagen, Tokyo, Japan) and stored at 4 °C.

### Genotyping and haplotype prediction

Genotyping of *AGXT2* and *DDAH1* SNPs was conducted using the TaqMan 5′-exonuclease allelic discrimination assay (*AGXT2*: Assay IDs: rs37369; C___11162986_1, rs37370; C__1018750_1, rs180749; C__1018795_1, and rs16899974; C__25742181_10, respectively, and *DDAH1*: Assay IDs: rs307894; C___2518300_20, rs669173; C__658778_10, rs997251; C__2518388_10, and rs13373844; C__1406532_10, respectively)] using the StepOnePlus real-time PCR system (Applied Biosystems, Foster City, CA). In addition, 4-nucleotide (GCGT) insertion/deletion (4 N ins/del) on the promoter region of *DDAH1* was determined by a custom TaqMan probe assay, as previously described (20167924). The haplotype within rs37370, rs37369, rs180749, and rs16899974 was predicted by SNPAlyze (Dynacom, Tokyo, Japan), which could estimate the individual diplotype [18].

### ADMA concentrations

An enzyme-linked immunoassay (ADMA Xpress ELIS; KR7860, Immundiagnostik, UK) was used to measure ADMA concentrations according to the manufacturer’s protocol.

### Statistical analysis

The Kolmogorov–Smirnov test of normality was conducted to determine whether the distribution was non-normal. The average difference among the two groups was tested by Student’s *t*-test or the Mann–Whitney *U* test, and the average difference among the three groups was tested by one-way analysis of variance (ANOVA) or the Kruskal–Wallis test according to the normality. In addition to these analyses, multiple regression analysis and analysis of covariance (ANCOVA) were conducted using SPSS 22.0 (IBM Japan, Tokyo, Japan). Multicollinearity was determined according to the following criterion: a variance inflation factor > 10. D′ for considering linkage disequilibrium (LD) was calculated by SNPAlyze (Dynacom), and the LD between two SNPs was set as more than D′ = 0.9. The estimations of the Hardy–Weinberg equilibrium (HWE) and minor allele frequency were conducted using Haploview software (version 4.2; Cambridge, MA, USA). Statistical significance was set at the 95% level (*p* = 0.05). The missing values for genotyping analysis were calculated by omission.

## Results

### Subjects recruited from the Nakayama study

The demographic data and biochemical data are shown in Tables 1 and 2, respectively. Because the participants were older, high rates of lifestyle diseases were found (e.g., hypertension: 560/749, 74.8%; DM: 120/743, 16.2%; kidney disease: 171/750, 22.8%).

**Table 1:** Demographic and biochemical data from the Nakayama study

Parameters	Available number	Values
**Demographic data**		
N		750
Age (years)	750	77.0 ± 7.6
Sex (male:female)	750	309:441
Height (cm)	746	151.7 ± 9.6
Weight (kg)	747	54.6 ± 10.6
BMI (kg/m^2^)	746	23.6 ± 3.3
Education (years)	743	
1: < 6		1: 33
2: 7–9		2: 324
3: 10–12		3: 323
4: ≥13		4: 63
Systolic blood pressure (mmHg)	749	137.8 ± 15.6
Diastolic blood pressure (mmHg)	749	76.7 ± 9.9
Heart rate (per min)	748	70.3 ± 12.0
Hypertension	749	560
Diabetes mellitus	743	120
Liver disease	748	44
Kidney disease	750	171
Depression	748	87
Brain attack (past history of stroke)	748	78
Head injury	748	37
Current alcohol drinking	724	235
Current smoking status	723	32

**Table 2:** Demographic and biochemical data from the Nakayama study

Parameters	Available number	Values
**Biochemical data**		
Albumin (g/dL)	750	4.19 ± 0.34
Total bilirubin (mg/dL)	750	0.72 ± 0.26
AST (IU/L)	750	25.5 ± 8.51
ALT (IU/L)	750	18.9 ± 11.7
LDH (IU/L)	750	220.0 ± 40.3
γ-GTP (IU/L)	750	29.8 ± 37.3
CPK	750	121.1 ± 70.5
Total cholesterol (mg/dL)	750	196.7 ± 31.8
LDL cholesterol (mg/dL)	750	110.8 ± 26.7
HDL cholesterol (mg/dL)	750	59.0 ± 26.7
Fasting blood glucose (mg/dL)	24	102.1 ± 42.0
Casual blood glucose (mg/dL)	726	117.4 ± 45.6
HbA1c (%)	750	5.85 ± 0.82
BUN (mg/dL)	750	17.2 ± 4.93
Creatinine (mg/dL)	750	0.76 ± 0.26
eGFR (mL/min/1.73m^2^)	750	66.7 ± 12.0
Na (mEq/L)	750	140.8 ± 2.32
K (mEq/L)	750	5.08 ± 0.67
Cl (mEq/L)	750	102.9 ± 2.68

*ALT* alanine aminotransferase, *AST* aspartate transaminase, *BMI* body mass index, *BUN* blood urea nitrogen, *CPK* creatine phosphokinase, *eGFR* estimated glomerular filtration rate, *γ-GTP* γ-glutamyl transpeptidase, *HDL* high density lipoprotein, *LDH* lactate dehydrogenase, *LDL* low-density lipoprotein

### Genotyping and haplotype analysis

The genotyping rates were > 90.0% across *AGXT2* and *DDAH1* SNPs, and none of the *p* values for the HWE reached statistical significance (Supplemental Table 1). In terms of LD, each pair of the four SNPs in *AGXT2* was not in linkage equilibrium when considering D′ values. However, the following five pairs in *DDAH1* had linkage equilibrium, as shown in Supplemental Table 2: rs3087894 and rs669173, D′ = − 1; rs3087894 and rs997251, D′ = 0.9961; rs3087894 and rs13373844, D′ = − 1; rs669173 and rs997251, D′ = − 0.9089; and rs669173 and rs13373844, D′ = 0.9171. Therefore, only rs997251 and rs13373844 within *DDAH1* SNPs were used in the multiple regression analysis. As a result of the predicted haplotype of *AGXT2* SNPs (rs37370, rs37369, rs180749, rs16899974), the highest rate was found in the CAAA haplotype (0.2429; Supplemental Table 3), which was the same finding as that in our previous study [7].

### Urinary excretion of D-AIB and plasma ADMA concentration

None of the 85 subjects had two CAAC haplotypes. Therefore, the average difference was tested between two groups (number of CAAC haplotypes = 0 or 1). Urinary R-3-AIB excretion was significantly higher in subjects with one CAAC haplotype than in those without a CAAC haplotype (*p* = 0.004; Supplemental Figure 1).

**Fig. 1:**
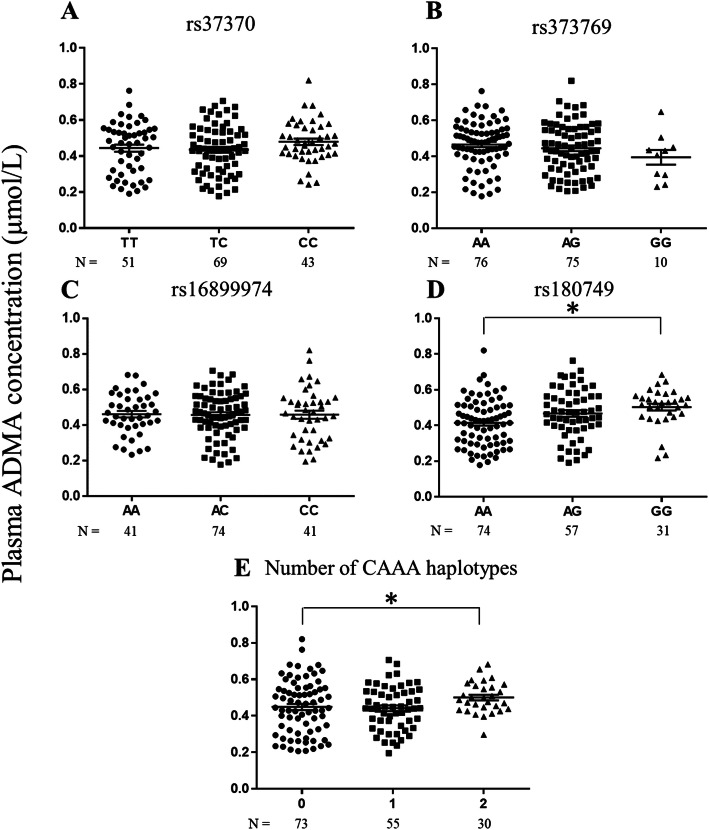
Effects of functional SNPs and CAAA haplotype in AGXT2 on ADMA concentrations. Average ADMA concentrations were tested by one-way ANOVA or the Kruskal–Wallis test among the **a** rs37370 (*p* = 0.222), **b** rs37369 (*p* = 0.238), **c** rs16899974 (*p* = 0.986), **d** rs180749 (*p* = 0.001), and **e** CAAA haplotypes (*p* = 0.083). The horizontal bar represents the mean ± standard error. Statistical significance based on a post hoc test among the two groups is indicated by an asterisk (*). CAAA was predicted by each allele of the four SNPs as follows: rs37370 (**c**), rs37369 (**a**), rs180749 (**a**), and rs16899974 (**a**). SNP, single nucleotide polymorphism

Plasma ADMA concentrations were significantly elevated in the AA genotype of rs187049 compared with the GG genotype (*p* = 0.001; overall *p* value = 0.001), and in subjects with two CAAA haplotypes compared with those without a CAAA haplotype (*p* = 0.033; overall *p* value = 0.083), as shown in Fig. 1. No significant changes were observed in rs37370 (*p* = 0.222), rs37369 (*p* = 0.238), or rs16899974 (*p* = 0.986). Additionally, no significant changes were found in *DDAH1* variants (rs997251, *p* = 0.559; rs13373844, *p* = 0.395; 4 N ins/del, *p* = 0.503), as shown in Supplemental Figure 2.

### Multiple regression analysis and ANCOVA

The details of each parameter used in the multiple regression analysis are explained in Supplemental Table 4.

### Systolic blood pressure (SBP) and diastolic blood pressure (DBP)

The number of CAAA haplotypes in *AGXT2* was significantly associated with both SBP (*p* = 0.031) and DBP (*p* = 0.028), as shown in Tables 3 and 4. In addition, rs16899974 was significantly associated with DBP (*p* = 0.040). Other than the *AGXT2* and *DDAH1* genotypes, the following parameters were significantly associated with SBP (sex [*p* <  0.001], body mass index [BMI; *p* = 0.020], depression [*p* = 0.026], head injury [*p* = 0.022], and total cholesterol [*p* = 0.003]) and with DBP (age [*p* = 0.03], sex [*p* = 0.002], BMI [*p* = 0.002], kidney disease [*p* = 0.004], depression [*p* = 0.042], and total cholesterol [*p* = 0.026]). The individual SNPs of *DDAH1* was not significantly associated with SBP and DBP (Supplemental Table 5a and 5b). The average differences in SBP and DBP among the number of CAAA haplotypes (0, 1, or 2) were tested by setting all values except the number of CAAA haplotypes as covariates. Both SBP (*p* = 0.546) and DBP (*p* = 0.767) showed a trend, but did not reach the level of statistical significance. The average DBP value was not significantly changed among the rs16899974 genotype (*p* = 0.431). Subsequently, we conducted liner regression analysis in non-HT and HT subjects separately with including antihypertensive drugs to investigate how AGXT2 SNPs and haplotype affect SBP and DBP when considering disease state and drugs affect. For SBP, the number of CAAA haplotypes showed a trend, but did not reach the level of statistical significance (*p* = 0.059) in HT subjects. In term of DBP, there were significant associations in rs16899974 (*p* = 0.009) and the number of CAAA haplotypes (*p* = 0.008) in HT subjects (Supplemental Table 6a and 6b).

**Table 3:** Multiple regression analysis for systolic blood pressure within *AGXT2* SNPs and haplotype

Parameters	β	*P* value
rs37370	− 0.081	0.17
rs37369	0.036	0.45
rs180749	0.041	0.36
rs16899974	0.047	0.38
CAAA haplotype	0.154	0.031
Age	0.082	0.075
Sex	−0.174	< 0.001
BMI	0.088	0.020
Education	0.017	0.69
Diabetes mellitus	0.069	0.069
Kidney disease	−0.019	0.64
Depression	−0.083	0.026
Brain attack	−0.023	0.54
Head injury	−0.086	0.022
Current alcohol drinking	0.042	0.31
Current smoking status	−0.055	0.17
Total cholesterol	0.119	0.003

*BMI* body mass index, brain attack (defined as past history of stroke)

**Table 4:** Multiple regression analysis for diastolic blood pressure within *AGXT2* SNPs and haplotype

Parameters	β	*P* value
rs37370	−0.065	0.26
rs37369	0.060	0.20
rs180749	−0.034	0.43
rs16899974	0.109	0.040
CAAA haplotype	0.155	0.028
Age	−0.096	0.034
Sex	−0.127	0.002
BMI	0.114	0.002
Education	0.031	0.45
Diabetes mellitus	−0.072	0.054
Kidney disease	−0.115	0.004
Depression	−0.075	0.042
Brain attack	0.014	0.71
Head injury	−0.061	0.098
Current alcohol drinking	0.039	0.33
Current smoking status	−0.022	0.58
Total cholesterol	0.087	0.026

*BMI* body mass index, brain attack (defined as past history of stroke)

### Aspartate aminotransferase (AST) and alanine aminotransferase (ALT)

The rs16899974 genotype was significantly associated with AST (*p* = 0.026) and ALT (*p* = 0.039), as shown in Tables 5 and 6. Furthermore, the number of CAAA haplotypes was significantly associated with AST (*p* = 0.011), but not with ALT (*p* = 0.083). Other than the *AGXT2* and *DDAH1* genotypes, the following parameters were significantly associated with AST (hypertension [*p* = 0.029]) and ALT (age [*p* <  0.001], BMI [*p* = 0.004], DM [*p* = 0.005], and depression [*p* <  0.001]). The individual SNPs of *DDAH1* was not significantly associated with AST and ALT (Supplemental Table 7a and 7b). The average differences in AST and ALT among the number of CAAA haplotypes and the rs16899974 genotype were tested by setting all values as covariates except for the number of CAAA haplotypes and rs16899974, respectively. No significant changes were seen for AST (rs1689974, *p* = 0.847; CAAA haplotype *p* = 0.300) or ALT (rs16899974, *p* = 0.915).

**Table 5:** Multiple regression analysis for AST within *AGXT2* SNPs and haplotype

Parameters	β	*P* value
rs37370	0.044	0.46
rs37369	−0.032	0.50
rs180749	−0.18	0.69
rs16899974	−0.122	0.026
CAAA haplotype	−0.185	0.011
Age	−0.066	0.16
Sex	−0.038	0.36
BMI	−0.027	0.49
Education	−0.007	0.88
Hypertension	0.084	0.029
Diabetes mellitus	0.036	0.35
Kidney disease	−0.018	0.66
Depression	0.071	0.062
Current alcohol drinking	0.025	0.55
Current smoking status	0.030	0.47
Total cholesterol	−0.054	0.19

*AST* aspartate aminotransferase, *BMI* body mass index

**Table 6:** Multiple regression analysis for ALT within *AGXT2* SNPs and haplotype

Parameters	β	*P* value
rs37370	0.023	0.68
rs37369	0.050	0.27
rs180749	0.007	0.87
rs16899974	−0.108	0.039
CAAA haplotype	−0.119	0.083
Age	−0.161	< 0.001
Sex	−0.063	0.11
BMI	0.107	0.004
Education	0.041	0.32
Hypertension	0.071	0.052
Diabetes mellitus	0.103	0.005
Kidney disease	−0.048	0.22
Depression	0.147	< 0.001
Current alcohol drinking	−0.010	0.81
Current smoking status	0.069	0.079
Total cholesterol	−0.032	0.41

*ALT* alanine aminotransferase, *BMI* body mass index

### Blood urea nitrogen (BUN) and creatinine

The rs37370 genotype was significantly associated with BUN (*p* = 0.020) and creatinine (*p* = 0.023), as shown in Tables 7 and 8. The rs180749 genotype also reached a significant level in BUN (*p* = 0.032). Other than the *AGXT2* and *DDAH1* genotypes, the following parameters were significantly associated with BUN (age [*p* <  0.001]) and creatinine (age [*p* <  0.001], sex [*p* <  0.001], and BMI [*p* = 0.014]). The individual SNPs of *DDAH1* was not significantly associated with BUN and creatinine (Supplemental Table 8a and 8b). The average differences in BUN among the rs37370 and rs180749 genotypes were tested by setting all values as covariates except the rs37370 and rs180749 genotypes, respectively. The average BUN value was not significantly changed in the rs37370 genotype (*p* = 0.675) and the rs180749 genotype (*p* = 0.158).

**Table 7:** Multiple regression analysis for BUN within *AGXT2* SNPs and haplotype

Parameters	β	*P* value
rs37370	0.134	0.020
rs37369	0.025	0.59
rs180749	0.094	0.032
rs16899974	0.039	0.46
CAAA haplotype	−0.063	0.37
Age	0.294	< 0.001
Sex	−0.060	0.14
BMI	−0.008	0.82
Education	0.069	0.097
Hypertension	−0.012	0.75
Diabetes mellitus	0.008	0.82
Liver disease	0.009	0.81
Depression	−0.046	0.21
Current alcohol drinking	0.011	0.78
Current smoking status	−0.020	0.62
Total cholesterol	0.003	0.93

*BMI* body mass index, *BUN* blood urea nitrogen

**Table 8:** Multiple regression analysis for creatinine within *AGXT2* SNPs and haplotype

Parameters	β	*P* value
rs37370	0.119	0.023
rs37369	−0.002	0.97
rs180749	0.011	0.79
rs16899974	0.028	0.56
CAAA haplotype	−0.070	0.27
Age	0.234	< 0.001
Sex	−0.424	< 0.001
BMI	0.084	0.014
Education	0.005	0.89
Hypertension	0.043	0.20
Diabetes mellitus	−0.031	0.35
Liver disease	0.037	0.28
Depression	−0.035	0.30
Current alcohol drinking	0.062	0.091
Current smoking status	−0.051	0.16
Total cholesterol	−0.018	0.61

*BMI* body mass index

### Casual blood sugar (CBS)

The rs16899974 genotype was significantly associated with CBS (*p* = 0.028), as shown in Table 9. The number of CAAA haplotypes showed a trend, but did not reach the level of statistical significance (*p* = 0.086). Other than the *AGXT2* and *DDAH1* genotypes, BMI was significantly associated with CBS (*p* = 0.013). The individual SNPs of *DDAH1* was not significantly associated with CBS (Supplemental Table 9). The average difference in CBS for the rs16899974 genotype was tested by setting all values as covariates except the rs1689974 genotype. The average CBS value showed a trend for the rs16899974 genotype, but did not reach the level of statistical significance (*p* = 0.310). Subsequently, we conducted liner regression analysis in non-DM and DM subjects separately with including antidiabetic drugs to investigate how AGXT2 SNPs and haplotype affect CBS when considering disease state and drugs affect. There were significant associations in rs16899974 (*p* = 0.026) and the number of CAAA haplotypes (*p* = 0.007) in non-DM subjects (Supplemental Table 10).

**Table 9:** Multiple regression analysis for casual blood sugar within *AGXT2* SNPs and haplotype

Parameters	β	*P* value
rs37370	0.007	0.91
rs37369	−0.035	0.47
rs180749	0.007	0.88
rs16899974	−0.122	0.028
CAAA haplotype	−0.126	0.086
Age	−0.033	0.46
Sex	−0.026	0.53
BMI	0.097	0.013
Education	−0.009	0.84
Hypertension	0.033	0.39
Liver disease	< 0.001	0.99
Depression	0.006	0.87
Current alcohol drinking	0.071	0.088
Current smoking status	0.073	0.078
Total cholesterol	−0.055	0.18

*BMI* body mass index

## Discussion

To our knowledge, this is the first study to report an association between the *AGXT2* genotype and both BP and biochemical data. The recruitment method used in this study was the census method (within those aged > 65 years), which aimed to measure all members of the whole target population in Nakayama town. The advantage of the census method is its accuracy in terms of recruiting a pure population as compared with a sampling method using each unit of the population because the effect of community background is theoretically excluded. We recruited a total of 927 subjects (61.3% of the whole population aged > 65 years). Among the subjects’ DNA samples, the genotypes of four functional SNPs in *AGXT2* and associated haplotypes were successfully measured, and the results, such as allele frequencies, were almost the same as those in our previous studies [5, 7]. In terms of *DDAH1* SNPs and 4 N ins/del, the allele frequencies of these genotypes were also similar to those in the dbSNP database (https://www.ncbi.nlm.nih.gov/snp/) and a previous report [16]. In addition, subjects with two CAAA haplotypes had higher concentrations of ADMA than those without the CAAA haplotype. This result strongly matched that each allele (rs37370 = C, rs37369 = A, rs16899974 = A, and rs180749 = A) of the CAAA haplotype is relevant to the loss of function [5, 7], and subjects who have the CAAA haplotype showed high R-3-AIB excretion [7]. However, we have to consider that ADMA concentrations were significantly higher in the GG genotype (gain of function) than in the AA genotype (loss of function) of rs180749. Because rs180749 is one of the functional SNPs that regulates AGXT2 activity, it is hard to reach a definite conclusion without considering other functional SNPs. Therefore, we think that the CAAA haplotype is a stronger predictor of AGXT2 activity than are functional SNPs. Regarding *DDAH1*, a previous study reported elevated plasma ADMA concentrations in subjects with an insertion allele in 4 N ins/del [19]; however, no significant results were found in this study. As only three subjects in the present study had the ins/ins genotype, more subjects are needed to clarify this point. Based on these results and insights, four functional SNPs in *AGXT2* (rs37370, rs37369, rs180749, and rs16899974), the CAAA haplotype predicted by those four SNPs, two SNPs (rs997251 and rs13373844), and 4 N ins/del in *DDAH1*, were considered to affect BP and several biochemical tests after excluding *DDAH1* SNPs with LD.

The more subjects who had a CAAA haplotype, the higher SBP and DBP values were confirmed. Our previous study showed that the CAAA haplotype is associated with a loss of function, which was revealed by measuring urine R-3-AIB [7]. Contrary to the result regarding the CAAA haplotype when considering AGXT2 activity, the CC genotype of rs16899974 (gain of function) was associated with elevated DBP. Because the CAAC haplotype changed from the A allele to the C allele on rs16899974, it still plays a role in the loss of function, and the effect of the CAAA haplotype on AGXT2 activity is more effective than that on rs16899974. Considering that AGXT2 plays a role in metabolizing ADMA, a decrease in NO production is induced among people who have the CAAA haplotype. Indeed, decreased plasma NO was found in *Agxt2* KO mice, which also showed elevated plasma ADMA concentrations and mean arterial pressure as measured in the carotid artery [17]. Doğan et al. (2018) reported a correlation between serum ADMA levels and SBP [20]. In addition, it has been shown that high ADMA levels in plasma/serum are relevant to the exaggerated BP response to exercise [8], congestive heart failure [9], and coronary atherosclerosis [11], and the *AGXT2* genotypes are associated with atrial fibrillation and ischemic stroke [13] and coronary heart disease [14]. Collectively, we hypothesize that the loss of AGXT2 function induces elevated SBP and DBP through the dysregulation of the NOS/ADMA pathway, which means that we can estimate the risk of cardiovascular diseases by detecting *AGXT2* SNPs.

The loss of AGXT2 function predicted by the CAAA haplotype contributes to a decrease in AST. Conversely, the loss of AGXT2 function predicted by rs16899974 contributes to elevated AST and ALT. AGXT2 is mainly present in the mitochondria of hepatocytes [21]. A previous isotope study showed the existence of α-keto-δ-(N^G^,N^G^-dimethylguanidino) valeric acid (DMGV) in the liver, which is a metabolite of ADMA [22]. These lines of evidence suggest that metabolism from ADMA to DMVG occurs even in the liver. Growing evidence has shown that elevated plasma ADMA levels are found in patients with severe acute alcoholic hepatitis [23] and acute liver failure [24]. However, the functions of AGXT2 and ADMA in the liver are still unknown under both the normal condition and disease state.

Higher BUN values were found in the CC genotype of rs37370 (loss of function) and GG genotype of rs180749 (gain of function), which are discrepant results in terms of the predicted AGXT2 function. Within kidney tissues, AGXT2 is strongly expressed in the renal proximal tubular epithelium [21]. It has been shown that HNF4α, a nuclear transcription factor that can work by binding on promoter regions of *AGXT2*, is co-localized in the renal proximal tubular epithelium [21]. However, AGXT2 function itself is still unknown, even though AGXT2 expression is driven in kidney tissue.

Surprisingly, the loss of function of AGXT2 activity predicted by rs16899974 (AA genotype) was associated with elevated CBS. Several reports have shown that elevated ADMA concentrations are found in subjects with type 1 DM [25, 26], type 2 DM [12] and poor glycemic control [27]. Furthermore, the circulating level of ADMA is relevant to several DM-related complications, such as microvascular complications [28, 29], cardiovascular complications [30], and diabetic nephropathy [31]. Given that subjects who have the AA genotype show higher ADMA concentrations [32], the elevated CBS in the AA genotype of *AGXT2* is caused by changes in ADMA levels. It may be therefore possible to predict type 2 DM complications and whether CBS tends to rise easily in patients based on rs16899974.

This study had several limitations. First, the number of samples was relatively small as a population-based study, and there are some missing values. In this sense, type I error is not completely excluded. However, we used the census method to determine the clinical test items in a unit (Nakayama town) accurately, which is a strong advantage compared with the sampling method. Second, several discrepant results were found in the results of the biochemical tests on liver and kidney functions. A possible cause for this was that we could not determine AGXT2 activity by one SNP because four functional SNPs are assumed to regulate AGXT2 activity. In addition, AGXT2 functions in the liver and kidney are still unknown, even though these expressions were confirmed [21]. Lastly, we did not include information on what and how much the recruited subjects had eaten before blood sampling for CBS. Taken together, further studies with large samples and more detailed information on the subjects’ backgrounds are needed to clarify these points and reproducibility.

## Conclusions

In conclusion, we revealed that the loss of AGXT2 function predicted by the CAAA haplotype induces elevated ADMA concentrations. Elevated ADMA concentrations through the CAAA haplotype may increase SBP/DBP and CBS, respectively. Despite the fact that additional research is needed in terms of AGXT2 function in the kidney and liver, the four functional SNPs of AGXT2 and their haplotypes are useful tools for predicting hypertension, DM, and their related complications. When considering the loss of AGXT2 function depends on races, clinicians need to think through the genetic background in daily clinical practice.

## Supplementary Information

**Additional file 1: Figure S1.** Average change in R-3-AIB based on CAAC haplotype. Average urinary R-3-AIB excretion values between subjects with one and no CAAC haplotypes were tested using the Mann–Whitney *U* test (*p* = 0.004). The horizontal bar represents the mean ± standard error. Statistical significance is indicated by an asterisk (*). R-3-AIB, the R-form of 3-aminoisobutyrate; CAAC, was predicted by each allele of the four SNPs as follows: rs37370 (C), rs37369 (A), rs180749 (A), rs16899974 (C).

**Additional file 2: Figure S2.** Effects of DDAH1 variants in AGXT2 on ADMA concentrations. Average ADMA concentrations were tested by one-way ANOVA or the Kruskal–Wallis test among A) rs997251 (*p* = 0.559), B) rs13373844 (*p* = 0.395), and C) –396 4 N ins/del (*p* = 0.503). The horizontal bar represents mean ± standard error. Ins, insertion; del, deletion.

**Additional file 3: Table S1.** Main characteristics of the select tagging SNPs in AGXT2 and DDAH1. **Table S2.** Linkage disequilibrium on the SNP results for AGXT2 and DDAH1. **Table S3.** Haplotype analysis for AGXT2 SNPs. **Table S4.** Explanation of each parameters used in the demographic data and multiple regression analysis. **Table S5a.** Association between Systolic Blood Pressure and SNPs in DDAH1. **Table S5b.** Association between Diastolic Blood Pressure and SNPs in DDAH1. **Table S6a.** Multiple regression analysis for systolic blood pressure in non-HT subjects within AGXT2 SNPs and haplotype. **Table S6b**. Multiple regression analysis for diastolic blood pressure in non-HT subjects within AGXT2 SNPs and haplotype. **Table S7a.** Association between AST and SNPs in DDAH1. **Table S7b.** Association between ALT and SNPs in DDAH1. **Table S8a.** Association between BUN and SNPs in DDAH1. **Table S8b.** Association between creatinine and SNPs in DDAH1. **Table S9.** Association between CBS and SNPs in DDAH1. **Table S10.** Multiple regression analysis for casual blood sugar within AGXT2 SNPs and haplotype.

## Data Availability

The datasets used and/or analyzed during the current study are available from the corresponding author on reasonable request.

## Abbreviations

4 N ins/del: 4-nucleotide (GCGT) insertion/deletion
ADMA: Asymmetric dimethylarginine
ALT: Alanine aminotransferase
ANOVA: One-way analysis of variance
AGXT2: Alanine:glyoxylate aminotransferase 2
AST: Aspartate aminotransferase
BMI: Body mass index
BP: Blood pressure
BUN: Blood urea nitrogen
CBS: Casual blood sugar
DBP: Diastolic blood pressure
DDAH1: Diaminoarginine dimethylaminohydrolase 1
DM: Diabetes mellitus
DMGV: α-keto-δ--(NG,NG-dimethylguanidino) valeric acid
HWE: Hardy–Weinberg equilibrium
KO: Knockout
LD: Linkage disequilibrium
NO: Nitric oxide
NOS: Nitric oxide synthase
R-3-AIB: R-form of 3-aminoisobutyrate
SBP: Systolic blood pressure
SNP: Single nucleotide polymorphisms
