# Supplementary figures and images for: Effects of AGXT2 variants on blood pressure and blood sugar among 750 older Japanese subjects recruited by the complete enumeration survey method
Source: BMC Genomics. 2021 Apr 20;22:287. doi: 10.1186/s12864-021-07612-3 (PMC8059213; doi:10.1186/s12864-021-07612-3)

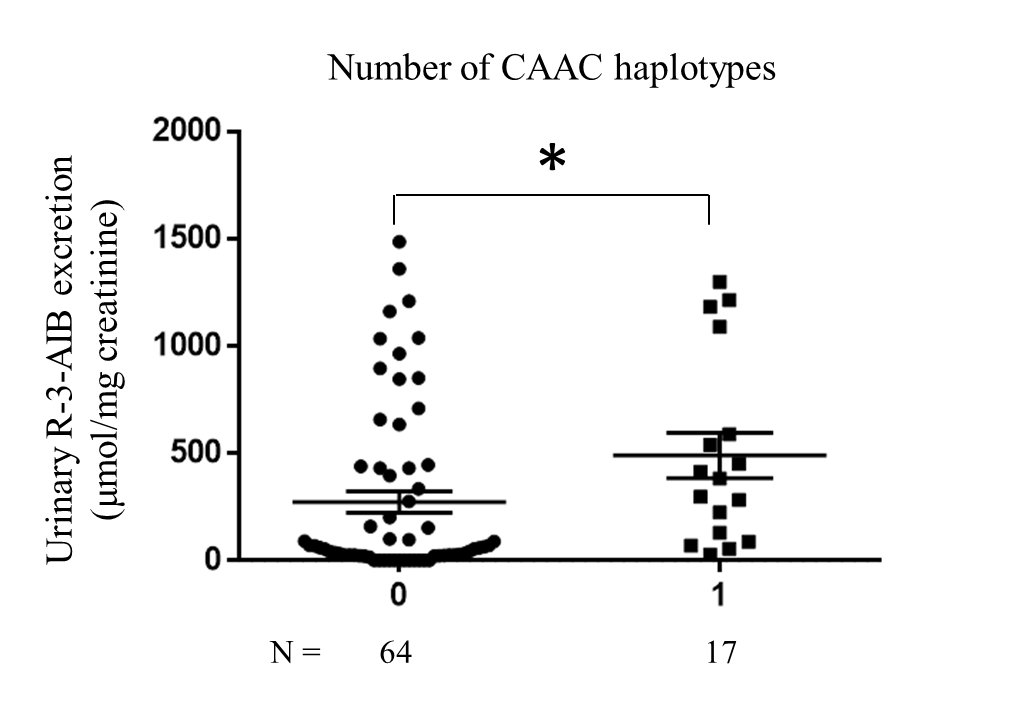

Supplement: Supplementary file 1 — Additional file 1: Figure S1. Average change in R-3-AIB based on CAAC haplotype. Average urinary R-3-AIB excretion values between subjects with one and no CAAC haplotypes were tested using the Mann–Whitney U test (p = 0.004). The horizontal bar represents the mean ± standard error. Statistical significance is indicated by an asterisk (*). R-3-AIB, the R-form of 3-aminoisobutyrate; CAAC, was predicted by each allele of the four SNPs as follows: rs37370 (C), rs37369 (A), rs180749 (A), rs16899974 (C). [file 12864_2021_7612_MOESM1_ESM.tif]

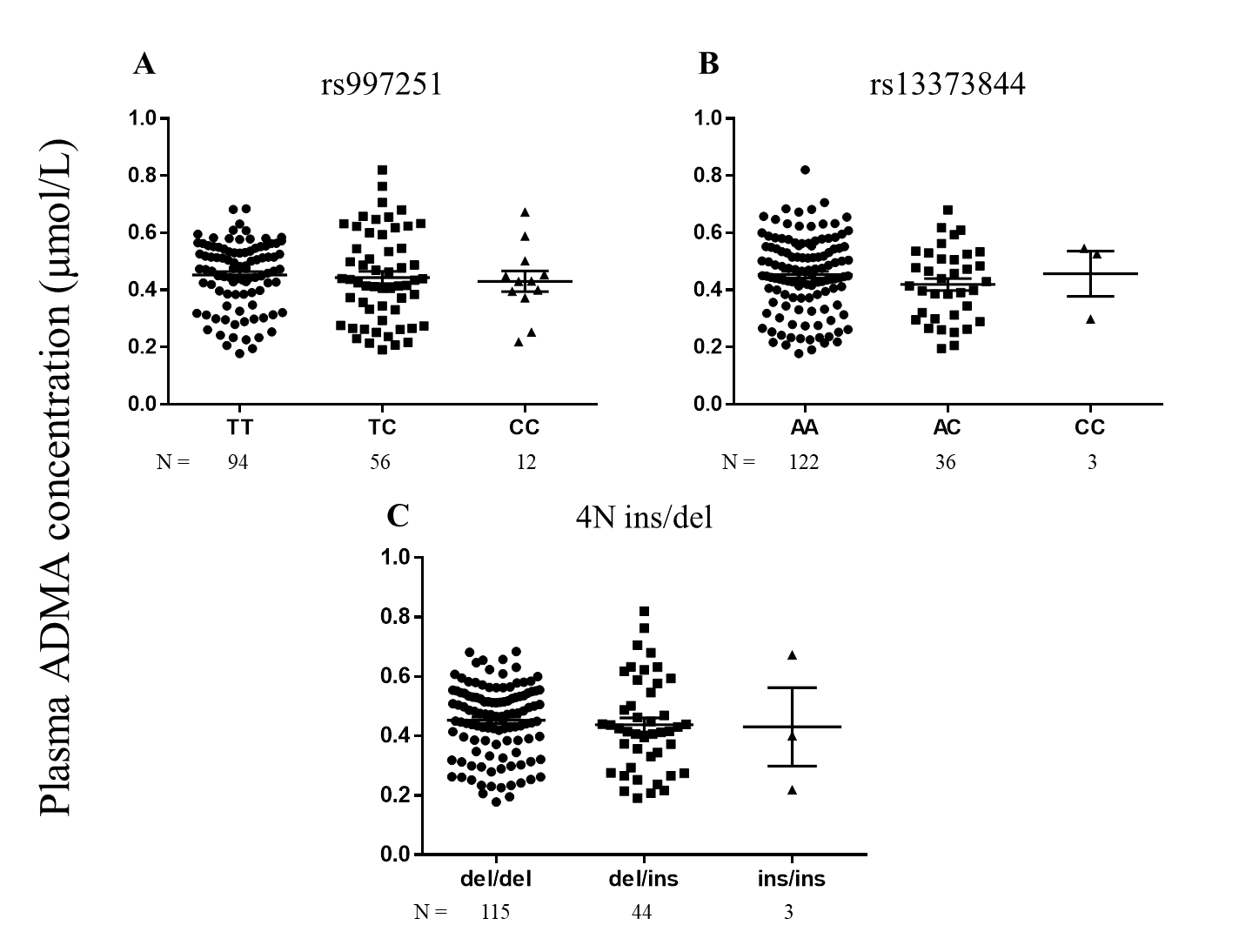

Supplement: Supplementary file 2 — Additional file 2: Figure S2. Effects of DDAH1 variants in AGXT2 on ADMA concentrations. Average ADMA concentrations were tested by one-way ANOVA or the Kruskal–Wallis test among A) rs997251 (p = 0.559), B) rs13373844 (p = 0.395), and C) –396 4 N ins/del (p = 0.503). The horizontal bar represents mean ± standard error. Ins, insertion; del, deletion. [file 12864_2021_7612_MOESM2_ESM.tif]
