# Supplementary material for: Effects of AGXT2 variants on blood pressure and blood sugar among 750 older Japanese subjects recruited by the complete enumeration survey method
Source: BMC Genomics. 2021 Apr 20;22:287. doi: 10.1186/s12864-021-07612-3 (PMC8059213; doi:10.1186/s12864-021-07612-3)
Supplement: Supplementary file 3 — Additional file 3: Table S1. Main characteristics of the select tagging SNPs in AGXT2 and DDAH1. Table S2. Linkage disequilibrium on the SNP results for AGXT2 and DDAH1. Table S3. Haplotype analysis for AGXT2 SNPs. Table S4. Explanation of each parameters used in the demographic data and multiple regression analysis. Table S5a. Association between Systolic Blood Pressure and SNPs in DDAH1. Table S5b. Association between Diastolic Blood Pressure and SNPs in DDAH1. Table S6a. Multiple regression analysis for systolic blood pressure in non-HT subjects within AGXT2 SNPs and haplotype. Table S6b. Multiple regression analysis for diastolic blood pressure in non-HT subjects within AGXT2 SNPs and haplotype. Table S7a. Association between AST and SNPs in DDAH1. Table S7b. Association between ALT and SNPs in DDAH1. Table S8a. Association between BUN and SNPs in DDAH1. Table S8b. Association between creatinine and SNPs in DDAH1. Table S9. Association between CBS and SNPs in DDAH1. Table S10. Multiple regression analysis for casual blood sugar within AGXT2 SNPs and haplotype. [file 12864_2021_7612_MOESM3_ESM.docx]

Effects of *AGXT2* variants on blood pressure and blood sugar among 750 older Japanese subjects recruited by the complete enumeration survey method

Yuta Yoshino^1^, Hiroshi Kumon^1^, Takaaki Mori^1^, Taku Yoshida^2^, Ayumi Tachibana^1^, Hideaki Shimizu^1^, Jun-ichi Iga^1^, Shu-ichi Ueno^1^

1. Department of Neuropsychiatry, Molecules and Function, Ehime University Graduate School of Medicine, Shitsukawa, Toon, Ehime 791-0295, Japan
2. Department of Neuropsychiatry, Zaidan Niihama Hospital, 13-47 Matsubara, Niihama, Ehime 792-0828, Japan

Supplementary Table 1. Main characteristics of the select tagging SNPs in *AGXT2* and *DDAH1*

| SNP (major/minor allele) | Localization | HWE p value |  | MAF | Genotyping rate (%) |
| --- | --- | --- | --- | --- | --- |
| AGXT2 |  |  |  |  |  |
| rs37370 (T/C) | exon 4 (V140I) | 0.97 |  | C (0.43) | 100 |
| rs37369 (A/G) | exon 3 (S102N) | 0.34 |  | G (0.38) | 99.87 |
| rs180749 (A/G) | exon 6 (T212I) | 0.26 |  | G (0.22) | 94.47 |
| rs16899974 (C/A) | exon 14 (V498L) | 0.42 |  | A (0.43) | 100 |
| DDAH1 |  |  |  |  |  |
| rs3087894 (T/A) | 3’-UTR | 0.35 |  | A (0.23) | 99.87 |
| rs669173 (T/C) | intron 4 | 0.32 |  | C (0.32) | 99.87 |
| rs997251 (T/C) | intron 5 | 0.23 |  | C (0.24) | 99.87 |
| rs13373844 (A/C) | intron 4 | 0.25 |  | C (0.12) | 99.87 |
| –396 4N INS/DEL | promoter |  |  | INS (0.16) | 99.87 |

DEL, deletion; INS, insertion

Supplementary Table 2. Linkage disequilibrium on the SNP results for AGXT2 and DDAH1

| AGXT2 | rs37370 | rs37369 | rs180749 | rs16899974 |
| --- | --- | --- | --- | --- |
| rs37370 |  |  |  |  |
| rs37369 | –0.6575 |  |  |  |
| rs180749 | –0.8535 | –0.2312 |  |  |
| rs16899974 | 0.2796 | –0.4221 | –0.4158 |  |
|  |  |  |  |  |
| DDAH1 | rs3087894 | rs669173 | rs997251 | rs13373844 |
| rs3087894 |  |  |  |  |
| rs669173 | –1 |  |  |  |
| rs997251 | 0.9961 | –0.9089 |  |  |
| rs13373844 | –1 | 0.9171 | –0.875 |  |

Supplementary Table 3. Haplotype analysis for AGXT2 SNPs

| rs37370 | rs37369 | rs180749 | rs168899 | Frequency |
| --- | --- | --- | --- | --- |
| C | A | A | A | 0.2429 |
| T | G | A | C | 0.2046 |
| C | A | A | C | 0.1331 |
| T | A | G | C | 0.1147 |
| T | G | A | A | 0.0721 |
| T | A | A | A | 0.048 |
| T | A | G | A | 0.0419 |
| T | A | A | C | 0.0398 |
| T | G | G | C | 0.0362 |
| C | G | A | C | 0.0303 |
| T | G | G | A | 0.0118 |
| C | G | A | A | 0.0117 |
| C | G | G | C | 0.00975 |
| C | A | G | A | 0.00238 |
| C | A | G | C | 0.00073 |
| C | G | G | A | 2.1E-16 |

Supplementary Table 4. Explanation of each parameters used in the demographic data and multiple regression analysis

| Parameters | Criteria | Values | Remarks |
| --- | --- | --- | --- |
| Sex |  | 0 = male, 1 = female |  |
| Hypertension | SBP ≥140 or DBP ≥90 or participants who were taking antihypertensive drugs | 0 = normal, 1 = hypertension | SBP and DBP were measured three times, and the average values were used. |
| Diabetes mellitus | FBS ≥126 or CBS ≥200 or HbA1c ≥6.5 or participants who were taking antidiabetic drugs | 0 = normal, 1 = diabetes mellitus | Based on ADA 2010 criteria |
| Liver disease | Past history of liver disease | 0 = none, 1 = yes | Based on interview |
| Depression | Past history of depression | 0 = none, 1 = yes | Based on interview |
| Brain attack | Past history of brain attack | 0 = none, 1 = yes | Based on interview |
| Head injury | Past history of head injury | 0 = none, 1 = yes | Based on interview |
| eGFR | Male: eGFR=0.813*141*min(creatinine/0.9, 1)^(–0.411)*max(creatinine /0.9, 1)^(–1.209)*0.993^Age  Female: eGFR=0.813*141*min(creatinine /0.7, 1)^(–0.329)*max(creatinine /0.7, 1)^(–1.209)*0.993^Age*1.018 |  | CKD epi formula adjusted for Japanese |
| Kidney disease | eGFR <60 | 0 = none, 1 = yes |  |
| rs37369 | Missense variant (T/C), located on exon 3 (S102N) | 1 = AA, 2 = AG, 3 = GG | A allele is associated with loss of function of AGXT2 |
| rs37370 | Missense variant (A/G), located on exon 4 (V140I) | 1 = TT, 2 = TC, 3 = CC | C allele is associated with loss of function of AGXT2 |
| rs180749 | Missense variant (A/G), located on exon 6 (T212I) | 1 = AA, 2 = AG, 3 = GG | A allele is associated with loss of function of AGXT2 |
| rs16899974 | Missense variant (C/A), located on exon 14 (V498L) | 1 = AA, 2 = AC, 3 = CC | A allele is associated with loss of function of AGXT2 |
| CAAA haplotype | Predicted by SNPAlyze software using rs37369, rs37370, rs180749, and rs16899974 | 0 = none, 1 = 1 haplotype, 2 = 2 haplotypes | CAAA haplotype is associated with loss of function of AGXT2 |
| rs997251 | Intron variant (T/C), located on intron 5 |  |  |
| rs13373844 | Intron variant (A/C), located on intron 4 |  |  |
| –396 4N insertion | 4-nucleotide (GCGT) deletion/insertion variant on promoter | 1 = del/del, 2 = del/ins, 3 = ins/ins | Insertion variant inhibits the transcription of DDAH1 |

ADA, American Diabetes Association; brain attack (defined as past history of stroke), CBS, casual blood sugar; DBP, diastolic blood pressure; del, deletion; FBS, fasting blood sugar; ins, insertion; SBP, systolic blood pressure.

Supplementary Table 5a. Association between Systolic Blood Pressure and SNPs in *DDAH1*

| SNPs | Association with systolic BP | | | | | |
| --- | --- | --- | --- | --- | --- | --- |
|  | Univariate | | | Multivariate* | | |
|  | β | S.E. | P value | β | S.E. | P value |
| rs997251 | 0.008 | 0.920 | 0.817 | 0.013 | 0.919 | 0.731 |
| rs13373844 | 0.025 | 1.224 | 0.492 | 0.031 | 1.206 | 0.401 |
| -396 4N ins/del | 0.002 | 1.073 | 0.948 | -0.010 | 1.091 | 0.790 |

BP, blood pressure; SNP, single-nucleotide polymorphism; S.E., standard error.

*Models accounted for sex, age, body mass index, level of education, diabetes mellitus, kidney diseases, depression, brain attack (defined as past history of stroke), head injury, alcohol intake, smoking status, and total serum cholesterol.

Supplementary Table 5b. Association between Diastolic Blood Pressure and SNPs in *DDAH1*

| SNPs | Association with diastolic BP | | | | | |
| --- | --- | --- | --- | --- | --- | --- |
|  | Univariate | | | Multivariate* | | |
|  | β | S.E. | P value | β | S.E. | P value |
| rs997251 | -0.018 | 0.585 | 0.617 | -0.012 | 0.585 | 0.738 |
| rs13373844 | -0.003 | 0.779 | 0.938 | 0.018 | 0.768 | 0.621 |
| -396 4N ins/del | -0.001 | 0.682 | 0.971 | -0.007 | 0.694 | 0.849 |

BP, blood pressure; SNP, single-nucleotide polymorphism; S.E., standard error.

*Models accounted for sex, age, body mass index, level of education, diabetes mellitus, kidney diseases, depression, brain attack (defined as past history of stroke), head injury, alcohol intake, smoking status, and total serum cholesterol.

Supplementary Table 6a. Multiple regression analysis for systolic blood pressure in non-HT subjects within *AGXT2* SNPs and haplotype.

|  | non-HT (n = 189) | | HT (n = 560) | |
| --- | --- | --- | --- | --- |
| parameters | β | P value | β | P value |
| rs37370 | 0.068 | 0.58 | -0.117 | 0.077 |
| rs37369 | 0.023 | 0.83 | 0.052 | 0.32 |
| rs180749 | -0.027 | 0.78 | 0.039 | 0.43 |
| rs16899974 | -0.136 | 0.21 | 0.063 | 0.30 |
| CAAA haplotype | 0.032 | 0.82 | 0.155 | 0.059 |
| Age | -0.072 | 0.78 | 0.048 | 0.14 |
| Sex | -0.239 | 0.007 | -0.179 | < 0.001 |
| BMI | -0.037 | 0.63 | 0.073 | 0.090 |
| Education | -0.001 | 0.99 | 0.007 | 0.89 |
| Diabetes mellitus | 0.072 | 0.35 | 0.070 | 0.10 |
| Kidney disease | 0.044 | 0.59 | -0.030 | 0.52 |
| Depression | -0.049 | 0.52 | -0.081 | 0.055 |
| Brain attack | -0.044 | 0.57 | -0.035 | 0.41 |
| Head injury | -0.127 | 0.11 | -0.056 | 0.18 |
| Current alcohol drinking | 0.022 | 0.79 | 0.039 | 0.40 |
| Current smoking status | -0.139 | 0.11 | -0.019 | 0.68 |
| Total cholesterol | 0.040 | 0.62 | 0.143 | 0.001 |
| Antihypertensive drugs |  |  | -0.240 | < 0.001 |

BMI, body mass index, brain attack (defined as past history of stroke)

Supplementary Table 6b. Multiple regression analysis for diastolic blood pressure in non-HT subjects within *AGXT2* SNPs and haplotype.

|  | non-HT (n = 189) | | HT (n = 560) | |
| --- | --- | --- | --- | --- |
| parameters | β | P value | β | P value |
| rs37370 | 0.167 | 0.18 | -0.118 | 0.062 |
| rs37369 | 0.066 | 0.53 | 0.079 | 0.11 |
| rs180749 | -0.066 | 0.50 | -0.044 | 0.35 |
| rs16899974 | -0.103 | 0.34 | 0.154 | 0.009 |
| CAAA haplotype | -0.106 | 0.46 | 0.209 | 0.008 |
| Age | -0.237 | 0.011 | -0.082 | 0.10 |
| Sex | -0.147 | 0.096 | -0.125 | 0.004 |
| BMI | -0.015 | 0.85 | 0.135 | 0.001 |
| Education | -0.054 | 0.54 | 0.030 | 0.51 |
| Diabetes mellitus | -0.017 | 0.83 | -0.089 | 0.030 |
| Kidney disease | 0.030 | 0.71 | -0.137 | 0.002 |
| Depression | -0.021 | 0.78 | -0.072 | 0.075 |
| Brain attack | -0.065 | 0.41 | 0.030 | 0.46 |
| Head injury | -0.053 | 0.51 | -0.045 | 0.27 |
| Current alcohol drinking | -0.070 | 0.41 | 0.065 | 0.14 |
| Current smoking status | -0.052 | 0.55 | 0.001 | 0.99 |
| Total cholesterol | 0.053 | 0.52 | 0.093 | 0.029 |
| Antihypertensive drugs |  |  | -0.245 | < 0.001 |

BMI, body mass index, brain attack (defined as past history of stroke); HT, hypertension

Supplementary Table 7a. Association between AST and SNPs in *DDAH1*

| SNPs | Association with diastolic AST | | | | | |
| --- | --- | --- | --- | --- | --- | --- |
|  | Univariate | | | Multivariate* | | |
|  | β | S.E. | P value | β | S.E. | P value |
| rs997251 | -0.029 | 0.503 | 0.434 | -0.031 | 0.532 | 0.405 |
| rs13373844 | -0.005 | 0.669 | 0.888 | -0.015 | 0.697 | 0.700 |
| -396 4N ins/del | -0.049 | 0.585 | 0.176 | -0.057 | 0.629 | 0.134 |

AST, aspartate aminotransferase; SNP, single-nucleotide polymorphism; S.E., standard error.

*Models accounted for sex, age, body mass index, level of education, hypertension, diabetes mellitus, kidney diseases, depression, alcohol intake, smoking status, and total serum cholesterol.

Supplementary Table 7b. Association between ALT and SNPs in *DDAH1*

| SNPs | Association with diastolic ALT | | | | | |
| --- | --- | --- | --- | --- | --- | --- |
|  | Univariate | | | Multivariate* | | |
|  | β | S.E. | P value | β | S.E. | P value |
| rs997251 | -0.040 | 0.689 | 0.278 | -0.049 | 0.695 | 0.177 |
| rs13373844 | -0.015 | 0.918 | 0.684 | -0.003 | 0.912 | 0.937 |
| -396 4N ins/del | -0.055 | 0.802 | 0.130 | -0.061 | 0.823 | 0.094 |

ALT, alanine aminotransferase ; SNP, single-nucleotide polymorphism; S.E., standard error.

*Models accounted for sex, age, body mass index, level of education, hypertension, diabetes mellitus, kidney diseases, depression, alcohol intake, smoking status, and total serum cholesterol.

Supplementary Table 8a. Association between BUN and SNPs in *DDAH1*

| SNPs | Association with diastolic BUN | | | | | |
| --- | --- | --- | --- | --- | --- | --- |
|  | Univariate | | | Multivariate* | | |
|  | β | S.E. | P value | β | S.E. | P value |
| rs997251 | 0.015 | 0.292 | 0.686 | 0.015 | 0.294 | 0.684 |
| rs13373844 | 0.007 | 0.388 | 0.850 | -0.005 | 0.386 | 0.902 |
| -396 4N ins/del | -0.021 | 0.340 | 0.575 | -0.020 | 0.349 | 0.591 |

BUN, blood urea nitrogen; SNP, single-nucleotide polymorphism; S.E., standard error.

*Models accounted for sex, age, body mass index, level of education, hypertension, diabetes mellitus, liver diseases, depression, alcohol intake, smoking status, and total serum cholesterol.

Supplementary Table 8b. Association between creatinine and SNPs in *DDAH1*

| SNPs | Association with diastolic creatinine | | | | | |
| --- | --- | --- | --- | --- | --- | --- |
|  | Univariate | | | Multivariate* | | |
|  | β | S.E. | P value | β | S.E. | P value |
| rs997251 | -0.024 | 0.016 | 0.508 | -0.004 | 0.014 | 0.893 |
| rs13373844 | 0.045 | 0.021 | 0.219 | 0.037 | 0.019 | 0.262 |
| -396 4N ins/del | -0.059 | 0.018 | 0.108 | -0.052 | 0.017 | 0.121 |

SNP, single-nucleotide polymorphism; S.E., standard error.

*Models accounted for sex, age, body mass index, level of education, hypertension, diabetes mellitus, liver diseases, depression, alcohol intake, smoking status, and total serum cholesterol.

Supplementary Table 9. Association between CBS and SNPs in *DDAH1*

| SNPs | Association with diastolic CBS | | | | | |
| --- | --- | --- | --- | --- | --- | --- |
|  | Univariate | | | Multivariate* | | |
|  | β | S.E. | P value | β | S.E. | P value |
| rs997251 | 0.037 | 2.728 | 0.314 | 0.041 | 2.852 | 0.281 |
| rs13373844 | -0.012 | 3.668 | 0.749 | -0.009 | 3.797 | 0.818 |
| -396 4N ins/del | 0.005 | 3.189 | 0.898 | 0.009 | 3.389 | 0.807 |

CBS, casual blood sugar; SNP, single-nucleotide polymorphism; S.E., standard error.

*Models accounted for sex, age, body mass index, level of education, hypertension, liver diseases, depression, alcohol intake, smoking status, and total serum cholesterol.

Supplementary Table 10. Multiple regression analysis for casual blood sugar within *AGXT2* SNPs and haplotype

|  | non-DM (n = 629) | | DM (n = 120) | |
| --- | --- | --- | --- | --- |
| parameters | β | P value | β | P value |
| rs37370 | 0.080 | 0.22 | -0.051 | 0.79 |
| rs37369 | -0.061 | 0.24 | -0.082 | 0.58 |
| rs180749 | -0.029 | 0.56 | -0.033 | 0.81 |
| rs16899974 | -0.135 | 0.026 | -0.244 | 0.12 |
| CAAA haplotype | -0.214 | 0.007 | -0.261 | 0.25 |
| Age | 0.029 | 0.55 | -0.116 | 0.37 |
| Sex | 0.053 | 0.24 | -0.056 | 0.64 |
| BMI | 1.117 | 0.007 | 0.049 | 0.64 |
| Education | -0.011 | 0.81 | -0.049 | 0.72 |
| Hypertension | 0.098 | 0.023 | -0.083 | 0.44 |
| Liver disease | 0.028 | 0.52 | -0.040 | 0.71 |
| Depression | 0.015 | 0.72 | 0.141 | 0.20 |
| Current alcohol drinking | 0.017 | 0.71 | 0.021 | 0.86 |
| Current smoking status | 0.049 | 0.28 | 0.061 | 0.60 |
| Total cholesterol | -0.007 | 0.88 | -0.082 | 0.47 |
| Antidiabetic drugs |  |  | -0.001 | 0.99 |

BMI, body mass index; DM, diabetes mellitus
